# Scraps

**Published:** 1892-03

**Authors:** 


### SCRAPS

PICKED UP BY THE ASSISTANT-EDITOR.

**German Measles.**—The books afford very little information as to the meaning of the word German in the term German measles. Under the word German, as related to Germany, the Sydenham Society's *Lexicon* and Dr. Billings' *National Medical Dictionary* give both German measles and German silver. This no doubt expresses the usual interpretation; but it seems more reasonable to suppose that in each of these cases the word is really "germane," from the same root as germ, and used in one case to characterise a disease which is closely allied to measles and in the other to denote an alloy which bears a resemblance to silver. If this is the case, the word is used as in the expression cousins-german, and should not have a capital letter for its initial. I shall be grateful if any reader will give me information on the point.

In reference to the term cousins-german, perhaps it is needless to say that at one time cousin was a word applied to any relative, and that cousins-german, or relatives closely allied, are those who are now usually called first cousins.

**Medical Philology** (I.)—Under this title I purpose from time to time citing the words having a medical significance which are to be found in the *Promptorium Parvulorum sive Clericorum, Dictionarius Anglo-Latinus princeps*, written about 1440, and in the *Catholicon Anglicum*, an English-Latin Word-book, dated 1483. These words will often afford additional instances of early authority for many modern expressions which now exist only in the vocabulary of the more ill-informed, and will also throw light on many an ancient medical theory or practice.

The *Promptorium* and the *Catholicon* have been issued by the Camden Society, and edited respectively by Mr. Albert Way and Mr. Sidney J. H. Herrtage, who have enriched the volumes by many learned annotations. There exist many MSS. and some early printed editions of the *Promptorium*. The Camden Society reprint is based upon the text of the Harleian MS. 221, but much use has been made of other versions, concerning which full particulars are given in the work The reprint of the *Catholicon* was made from a collation of a MS. belonging to Lord Monson and the British Museum Addit. MS., 15,562. The editors' notes I shall quote almost, if not quite, in full, and shall add any further illustrations I may be able to find; but, except in the case of the *New English Dictionary* now in course of publication, this will be rarely possible, as the notes are so full and illustrative. As dictionaries are frequently quoted in these notes and are cited by the names of their compilers, it will save much repetition if I give here the short titles and dates of these works:

PALSGRAVE ... *Lesclarcissement de la Langue Francoyse.* 1530. This contains an English-French Vocabulary. The book was reprinted in 1852 in Paris.

COOPER ... *Thesaurus Linguæ Romanæ et Britannicæ.* This, a revision of Sir Thomas Elyot's Latin and English Dictionary, was first published in 1532. Second and third editions were issued in 1552 and 1565.

LEVINS ... ... *Manipulus Vocabulorum.* 1570. This was reprinted by the Camden Society in 1867, and then described as "A Dictionary of English and Latin Words, arranged in the Alphabetical order of the last Syllables."

BARET ... ... *An Alvearie or Triple Dictionarie, in Englishe, Latin, and French.* 1573. In 1580, after the death of Baret, who was M.D. of Cambridge, a second edition was published, in which Greek was added to the other languages.

FLORIO ... ... *A Worlde of Wordes, Or Most copious, and exact Dictionarie in Italian and English.* 1598. A second edition was published in 1611.

COTGRAVE ... *A Dictionarie of the French and English Tongues.* This, exceedingly rich in English synonyms, appeared first in 1611. Other editions were published in 1632, 1650, 1660, and 1673.

MINSHEU ... The first edition of Minsheu's *Ductor in Linguas*, a polyglot dictionary dealing with eleven languages, was issued in 1617. A second edition was published in 1627.

HALLIWELL... *A Dictionary of Archaic and Provincial Words*, first published in 1847, reached a ninth edition in 1878.

WEDGWOOD... The first volume of his *Dictionary of English Etymology* was issued in 1859. Other volumes followed. There was a second edition of the complete work in 1872, and a third in 1878.

The British Museum MS. of the *Catholicon* has "An **Agnayll***e*," without any Latin equivalent. This word has a curious history. In the modern English rendering of the tenth century Saxon version of the Herbarium of Apuleius given in *Leechdoms, Wortcunning, and Starcraft of Early England*, edited in 1864 by the Rev. Oswald Cockayne, is the following (Vol. I., p. 147): "For the disorder the Greeks name παρωνυχίας, *angnails*, take root of this same wort [βολβὸς σκιλλητικός, bulb of scilla maritima], pound with vinegar and with a loaf, lay it to the sore; wonderfully it healeth the same."

Mr. Herrtage's note in the *Catholicon* is:

"A sore either on the foot or hand. Palsgrave has 'an *agnayle* upon one's too,' and Baret, 'an agnaile or little corn growing upon the toes, *gemursa, pterigium*.' Minsheu describes it as a 'sore betweene the finger and the nail.' '*Agassin.* A corne or agnele in the feet or toes. *Frouelle.* An agnell, pinne, or warnell in the toe.' 1611. Cotgrave. 'Agnayle: *pterigium*.' Manip. Vocab. According to Wedgwood 'the real origin is Ital. *anguinaglia* (Latin *inguem*), the groin, also a botch or blain in that place; Fr. *angonailles*. Botches, (pockie) bumps, or sores, Cotgrave.' Halliwell, *s.v.* quotes from the Med. MS. Lincoln, leaf 300, a receipt 'for *agnayls* one mans fete or womans.' Lyte, in his edition of Dodoens, 1578, p. 279, speaking of 'Git, or Nigella,' says:—'The same stieped in olde wine, or stale pisse (as Plinie saith) causeth the Cornes and *Agnayles* to fall of from the feete, if they be first scarified and scotched rounde aboute.' '*Gemursa.* A corn or lyke griefe vnder the little toe.' Cooper."

The Rev. A. S. Palmer, in *Folk-Etymology*, 1882, says:

"This word in all probability has nothing to do, as its present form would suggest, with the *nails* of the fingers (A. Sax. *angnägl* (?), pain-nail). It was formerly spelt *agnel, agnayle, angnayle*, and denoted a corn on the toe, or generally any hard swelling. It is doubtless the same word as Fr. *angonailles* . . . . . . It. *anguinaglia* . . . . . . 'also a disease in the inside of a horse's hinder legs,' (Florio). *Anguinaglia*, as Diez shows, is for *inguinalia*, a disease or affliction of *inguine*, Lat. *inguen*, the groin or flank (Sp. *engle*, Fr. *aine*). . . . . . . Turner, *Herbal* [1551], speaks of '*angnaylles* and such hard swellinges,' Florio of '*agnels*, wartles, almonds, or kernels growing behind the eares and in the necke' (s.v. *Páno*). 'The inner flesh or pulp [of a Gourd] is passing good for to be applied to the *agnels* or corns of the feet.' Holland, Pliny's Nat. Hist. ii. 36 (1634). '*Ghiandole, Agnels*, wartles, or kernels in the throat,' Florio."

Under the words quoted by Mr. Palmer from Florio, the word "agnels" does not occur in the 1598 edition.

The Rev. W. W. Skeat, in his *Etymological Dictionary*, goes fully into the history of the word. He connects the first syllable of the word with *angina* and ἀγχόνη, respectively from *angere* and ἄγχειν, to choke, and from the Aryan root AGH or ANGH, to choke, compress, afflict, and adds that "from the same root come *anger*, *anxious*, &c.; and the notion of 'inflamed' is often expressed by 'angry.' . . . . A corn would be called an *agnail* because caused by irritation or pressure. And from the same root must also come the first syllable of the A.S. *ang-nægl*."

The *New English Dictionary*, after giving various forms of the word, says that the application has been much perverted by pseudo-etymology, and points out that the root of the second syllable in the word is from the Gothic *nagls*, which had the sense, not of "finger-nail," *unguis*, but of a nail (of iron, etc.) *clavus*, which in Latin was both a nail in that sense and a corn in the foot. The syllable *nail* was referred subsequently to a finger- or toe-nail. It quotes from a passage in *Leechdoms* (Vol. II., p. 80), which, modernised, reads thus: "For an angnail, brass filings and old soap, and oil if thou have it, if thou have it not, add cream, mingle together, lay on;" and then gives the following additional instances of the word used to signify a corn:

"'Clauus is the latin . . . In englyshe it is named cornes or agnelles in a mannes fete or toes.' Boorde, *Breuiary* II. (1552) 3. '[Aloe] heleth also agnales when they are cut of.' Turner, *Herbal* (1568), 17. 'They skinne a kybed heele, they fret an angnale off.' Turberville, *Venerie* (1575), 137."

Under the equivalent of whitlow, the *New English Dictionary* gives from Lyte's *Dodoens*, 258: "Good to be layde unto . . . . ulcered nayles, or agnayles, whiche is a paynefull swelling aboute the ioyntes and nayles." But the word is given under its wrong definition, as here "agnayles" is not a synonym for "ulcered nayles," which could not be described as "a painful swelling about the joints and nails."

Thus it may be seen that till the 17th century there is no instance of the word agnail being used to denote anything else than a hard corn, and that probably Minsheu in 1617 was the first to chronicle its perverted meaning.

Occasionally in modern literature the word has become confounded with hangnails, sometimes popularly known as back-friends, being "small pieces of partially separated skin about the roots of the finger-nails." The *New English Dictionary* quotes from *Weldon's Illustrated Dress-maker*, 1882: "This method practised daily will keep the nails in perfect preservation, also preventing agnails." The Sydenham Society's *Lexicon*, in which better things might have been expected, contents itself with defining Agnail as "a term applied to the shreds of epidermis which separate from the skin covering the root of the nails, and which, on being torn, give rise to a painful state of the fingers." In smaller recent dictionaries the word appears perverted from its original meaning. Chambers defines it as "an inflammation around the nail." Nuttall, with less circumlocution, gives "a whitlow" as its equivalent.

It is clear that the earlier signification of the word (see the quotations from Palsgrave, Cooper, Baret, and others) had nothing whatever to do with a sore near the nails, or with a blotch in the groin, and that Mr. Cockayne was not right in giving *angnails* as a gloss upon παρωνυχίας, as at the date of the Saxon version of the *Herbarium* the word would not have denoted that condition, which was undoubtedly a whitlow. Neither is he justified in giving παρωνυχία as a marginal reading to "angnail" in the quotation cited from Vol. II. of *Leechdoms*, as the section in which the word occurs is one dealing with "warty eruptions," amongst which a corn would be classed. Mr. Herrtage's own definition of the word is not quite correct.

